# Association of Heart Rate With Troponin Levels Among Patients With Symptomatic Atrial Fibrillation

**DOI:** 10.1001/jamanetworkopen.2020.16880

**Published:** 2020-09-22

**Authors:** Jussi-Pekka Pouru, Samuli Jaakkola, Fausto Biancari, Tuomas O. Kiviniemi, Ilpo Nuotio, K.E. Juhani Airaksinen

**Affiliations:** 1 Heart Center, Turku University Hospital and University of Turku, Turku, Finland; 2 Research Unit of Cardiac Surgery, Anesthesia and Critical Care, University of Oulu, Oulu, Finland; 3 Heart and Lung Center, Helsinki University Hospital, Helsinki, Finland; 4 Department of Acute Internal Medicine, Turku University Hospital and University of Turku, Turku, Finland

## Abstract

This cohort study investigates heart rate and cardiac troponin levels in patients admitted to the emergency department with symptomatic atrial fibrillation.

## Introduction

Cardiac troponins are routinely used to rule in or rule out acute coronary syndrome in the emergency department (ED). Among patients with symptomatic atrial fibrillation (AF), mildly elevated troponin levels are common but rarely caused by type 1 myocardial infarction.^1^ We aimed to investigate the association of heart rate with high-sensitivity cardiac troponin T (hs-cTn T) levels in patients admitted to the ED for AF.

## Methods

The Ethics Committee of the Hospital District of Southwest Finland approved this cohort study and determined that written informed consent was not required because of the retrospective nature of the study. This study is reported following the Strengthening the Reporting of Observational Studies in Epidemiology (STROBE) guideline for the reporting of observational studies. The Tropo-AF study^1^ aimed to investigate the factors associated with minor hs-cTn T elevations (<0.1 ng/mL; to convert to micrograms per liter, multiply by 1) in 2911 patients with AF at the ED; 501 patients from the Tropo-AF study fulfilled the inclusion criteria for this cohort study: at least 2 hs-cTn T samples within 72 hours and principal discharge diagnosis of AF. The primary outcome measure was peak hs-cTn T serum level within 72 hours of ED admission. Categorical variables were compared using Fisher exact test or Mantel-Haenszel linear-by-linear association test for trend, and continuous variables were compared between groups using Mann-Whitney *U* test. *P* values were 2-sided, and significance was set at *P* < .05. Statistical evaluation was conducted from January 2020 to April 2020 using statistical software SPSS for Windows version 25.0 (IBM) and R version 3.6.1 (R Project for Statistical Computing) (eAppendix in the Supplement).

## Results

A total of 501 patients (median [interquartile range] age, 75.6 [66.6-82.3] years; 262 [52.3%] women) were included in analysis (Table). In multiple linear regression analysis (Akaike information criterion, 4319.44), increase in peak hs-cTn T level was independently associated with a higher heart rate (β = 0.194; *P* < .001). Other factors independently associated with peak hs-cTn T levels were age (β = 0.141; *P* = .004), low hemoglobin levels (β = 0.142; *P* = .001), decreasing estimated glomerular filtration rate (β = 0.130; *P* = .004), diabetes (β = 0.129; *P* = .001), heart failure (β = 0.124; *P* = .002), new-onset AF (β = 0.155; *P* < .001), and absence of palpitation (β = 0.152; *P* < .001).

**Table.** Baseline Characteristics and Follow-up Data

Characteristic	No. (%)			*P* value
	Overall (N = 501)	Heart rate, bpm		
		<125 (n = 299)	≥125 (n = 202)	
Age, median (IQR), y	75.6 (66.6-82.3)	76.0 (66.7-82.4)	75.0 (66.3-82.1)	.41
Women	262 (52.3)	142 (47.5)	120 (59.4)	.01
CHA_2_DS_2_-VASc score, median (IQR)	3 (2-4)	3 (2-4)	3 (2-4)	.43
Congestive heart failure	45 (9.0)	29 (9.7)	16 (7.9)	.53
Hypertension	318 (63.5)	192 (64.2)	126 (62.4)	.71
Diabetes mellitus	93 (18.6)	60 (20.1)	33 (16.3)	.35
Prior stroke/TIA	60 (12.0)	37 (12.4)	23 (11.4)	.78
Coronary artery disease^a^	131 (26.1)	85 (28.4)	46 (22.8)	.18
Prior myocardial infarction	67 (13.4)	42 (14.0)	25 (12.4)	.69
Prior PCI or CABG	66 (13.2)	37 (12.4)	29 (14.4)	.59
Hypercholesterolemia	193 (38.5)	119 (39.8)	74 (36.6)	.51
Current smoker	28 (5.6)	13 (4.3)	15 (7.4)	.17
Active malignancy	26 (5.2)	14 (4.7)	12 (5.9)	.54
Types of AF				
Permanent or persistent	91 (18.2)	69 (23.1)	22 (10.9)	.001
Paroxysmal	280 (55.9)	165 (55.2)	115 (56.9)	.72
New-onset	130 (25.9)	65 (21.7)	65 (32.2)	.01
Antithrombotic therapy				
Antiplatelet therapy	120 (24.0)	74 (24.7)	46 (22.8)	.67
Acetylsalicylic acid	108 (21.6)	70 (23.4)	38 (18.8)	.23
Clopidogrel	14 (2.8)	5 (1.7)	9 (4.5)	.10
Anticoagulation	269 (53.7)	173 (57.9)	96 (47.5)	.03
Antiarrhythmic agents	341 (68.1)	219 (73.2)	122 (60.4)	.003
β blockers	332 (66.3)	214 (71.6)	118 (58.4)	.003
Symptoms				
Chest pain	99 (19.8)	57 (19.1)	42 (20.8)	.65
Dyspnea	133 (26.5)	79 (26.4)	54 (26.7)	>.99
Palpitations	250 (49.9)	148 (49.5)	102 (50.5)	.86
Heart rate at admission, median (IQR), bpm^b^	116 (95-135)	98 (83-113)	138 (131-146)	<.001
Laboratory variables, median (IQR)				
Peak hs-cTn T at 72 h, ng/mL	0.020 (0.012-0.038)	0.018 (0.011-0.032)	0.026 (0.014-0.045)	<.001
Systolic blood pressure, mm Hg^c^	136 (120-150)	136 (121-152)	135 (118-149)	.29
Hemoglobin, g/dL	13.8 (12.5-14.8)	13.8 (12.5-14.9)	13.7 (12.6-14.7)	.68
C-reactive protein, mg/dL^d^	0.3 (0.2-1.0)	0.3 (0.2-0.8)	0.4 (0.2-1.1)	.01
eGFR, mL/min/1.73 m^2^^e^	66.6 (51.3-82.4)	66.1 (50.5-80.6)	67.8 (53.7-84.1)	.24
<60	186 (37.1)	115 (38.5)	71 (35.1)	.51
End points at 1 y				
All-cause mortality	41 (8.2)	19 (6.4)	22 (10.9)	.10
Myocardial infarction	4 (0.8)	3 (1.0)	1 (0.5)	.65
Stroke	4 (0.8)	4 (1.3)	0 (0)	.15

Abbreviations: AF, atrial fibrillation; bpm, beats per minute; CABG, coronary artery bypass graft; eGFR, estimated glomerular filtration rate; hs-cTn T, high-sensitivity cardiac troponin T; IQR, interquartile range; PCI, percutaneous coronary intervention; TIA, transient ischemic attack.

SI conversion factors: To convert hs-cTn T to micrograms per liter, multiply by 1; hemoglobin to grams per liter, multiply by 10; C-reactive protein to milligrams per liter, multiply by 10.

^a^ Defined as a diagnosis of coronary disease, history of myocardial infarction, percutaneous coronary intervention, or coronary artery bypass graft surgery.

^b^ Heart rate was determined from the first 12-lead electrocardiogram obtained during the emergency department visit.

^c^ Missing data on systolic blood pressure in 60 patients (12%).

^d^ Missing data on C-reactive protein in 85 patients (17%).

^e^ Calculated using the Chronic Kidney Disease Epidemiology Collaboration equation.

Multiple logistic regression revealed that the association of heart rate with elevated hs-cTn T level (>0.014 ng/mL) was limited to patients in the 2 groups with the highest heart rates (heart rate, 125-139 beats per minute [bpm]: adjusted odds ratio, 2.03; 95% CI, 1.05-3.90; *P* = .03; heart rate ≥140 bpm: adjusted odds ratio, 4.05; 95% CI, 1.80-9.12; *P* = .001) compared with patients with admission heart rate less than 90 bpm. The generalized additive model confirmed a nonlinear association between peak hs-cTn T and admission heart rate (Figure, A). While 68 of 85 patients with CHA_2_DS_2_-VASc score 4 or higher and heart rate 125 bpm or higher (80.0%) had elevated hs-cTn T levels, only 17 of 48 patients with CHA_2_DS_2_-VASc score at least 1 and heart rate less than 125 bpm (35.4%) had elevated hs-cTn T levels (P for overall trend < .001) (Figure, B).

**Figure.**
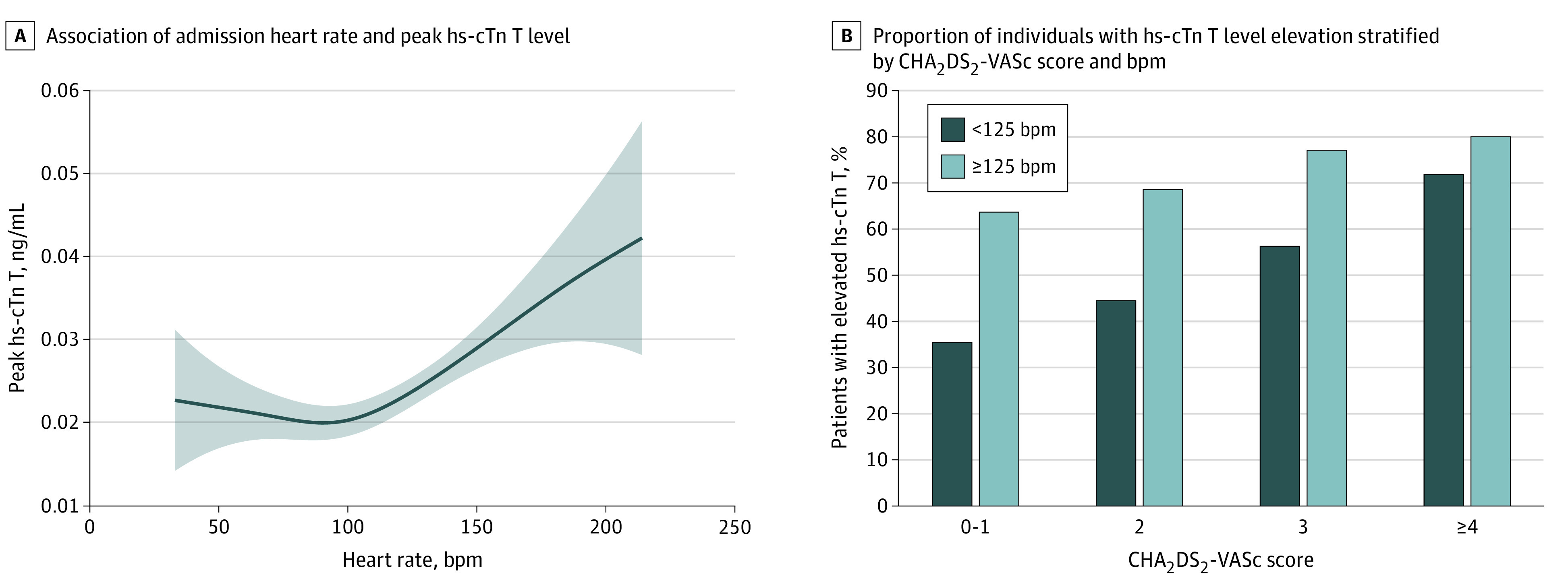
Association of High-Sensitivity Cardiac Troponin T (hs-cTn T) Level With Heart Rate A. Generalized additive model (adjusted *R*^2^ = 0.249; Akaike information criterion, 4232.21; estimated *df* = 3.04; *P* < .001). Adjustments were made for age, hemoglobin, estimated glomerular filtration rate, diabetes, congestive heart failure, new-onset atrial fibrillation, and palpitation symptoms. Shaded region indicates 95% CIs of the smooth function (continuous line) with the uncertainty of the overall mean. B. Elevated hs-cTn T was defined as higher than 0.014 ng/mL (to convert to micrograms per liter, multiply by 1). bpm indicates beats per minute.

Patients with coronary artery disease had higher median (interquartile range) peak hs-cTn T levels (0.024 ng/mL [0.014-0.045]) compared with patients without known disease (0.019 [0.011-0.035] ng/mL; *P* = .001). The peak hs-cTn T level was independently associated with higher heart rate in patients with known coronary artery disease (β = 0.168; *P* = .04) and those without (β = 0.205; *P* < .001; *P* for interaction = .66).

Of 501 patients, 69 patients (13.8%) had a dynamic change of more than 50% in hs-cTn T level. Multiple logistic regression analysis showed that heart rate 140 bpm or greater was significantly associated with a change of more than 50% in hs-cTn T level (adjusted odds ratio, 4.61; 95% CI, 1.62-13.14; *P* = .004).

## Discussion

This cohort study found that high ventricular heart rate was significantly associated with troponin release in patients admitted to the ED primarily for symptomatic AF. The association of heart rate with hs-cTn T release was nonlinear and became evident above the heart rate threshold of 125 bpm.

Persistent mild elevation of troponin levels is a common finding in AF, but the underlying etiopathogenesis remains unclear.^1,2^ Old age and multiple comorbidities were associated with minor troponin elevations in patients with AF independent of heart rate. This study’s findings suggest that inadequate ventricular rate control in the acute setting was associated with increased troponin T levels. Patients with elevated troponin levels were not at increased cardiovascular risk, and the magnitude of troponin release was not associated with the presence of known coronary artery disease or chest pain. Similarly, elevated troponin levels can be observed after marathon racing, supraventricular tachycardia, or rapid atrial pacing in individuals with normal coronary arteries and even in individuals without biochemical evidence of myocardial ischemia.^3,4,5,6^

Limitations of this study include its retrospective design, small sample size, and determination of heart rate from single admission electrocardiogram. Nevertheless, these results suggest that high ventricular heart rate at admission was independently associated with troponin release in patients with AF. The effect of ventricular heart rate should be taken into consideration in the differential diagnosis of patients with AF and increased troponin levels.

## Notes

Supplement. **eAppendix.** Supplementary Methods
