# Supplementary material for: Association of Heart Rate With Troponin Levels Among Patients With Symptomatic Atrial Fibrillation
Source: JAMA Netw Open. 2020 Sep 22;3(9):e2016880. doi: 10.1001/jamanetworkopen.2020.16880 (PMC7509607; doi:10.1001/jamanetworkopen.2020.16880)
Supplement: Supplement. — eAppendix. Supplementary Methods [file jamanetwopen-e2016880-s001.pdf]

Supplementary Online Content

Pouru JP, Jaakkola S, Biancari F, Kiviniemi TO, Nuotio I, Airaksinen KEJ. Association of heart rate with troponin level among patients with symptomatic atrial fibrillation. *JAMA Netw Open*. 2020;3(9):e2016880. doi:10.1001/jamanetworkopen.2020.16880

eAppendix. Supplementary Methods

This supplementary material has been provided by the authors to give readers additional information about their work.

© 2020 Pouru JP et al. *JAMA Network Open.*

eAppendix. Supplementary Methods

Tropo-AF study (ClinicalTrials.gov Identifier: NCT03683836) is a part of wider protocol in progress to assess clinical challenges in the treatment of atrial fibrillation (AF) in Western Finland. The main aim of this retrospective study is to investigate the etiology of minor (below 100 ng/L) high-sensitivity cardiac troponin T (hs-cTnT) elevations in AF patients admitted to the emergency department (ED) of the Turku University Hospital, Finland, between March 2013 and April 2016. The study was conducted in accordance with the seventh revision of the Declaration of Helsinki, and was approved by the Ethics Committee of the Hospital District of Southwest Finland. Written informed consent was not required because of the retrospective nature of this study. The manuscript was written following the STROBE guidelines for the reporting of observational studies. Data were evaluated from January 2020 to April 2020.

The first standard 12-lead electrocardiogram (ECG) on admission was used to confirm AF and to derive heart rate (HR). Among patients with multiple ED admissions during the study period, only the first one was considered for this analysis. Patients aged <18 years or residing outside the catchment area of Turku University Hospital were excluded from this study. Comprehensive clinical data were manually collected from individual electronic patient records using a standardized electronic case report form. Data on all-cause mortality, myocardial infarctions and ischemic strokes were collected from a period of 12 months for all these patients. The primary outcome measure of this study was peak hs-cTnT value within 72 hours of ED admission.

Laboratory data were collected through computerized search of the laboratory database provided by Turku University Hospital laboratory service (TYKSLAB, Turku, Finland). Throughout the study period, hs-cTnT samples were measured and analyzed using a commercial hs-cTnT assay (Elecsys Troponin T-high sensitive, Roche Diagnostics GmbH, Mannheim, Germany) in the hospital laboratory using standard methods. The limit of detection for this assay is 5 ng/L (to convert to micrograms per liter, multiply by .001), and the 99th percentile upper reference is 14 ng/L in a healthy reference population. Subjects with values below the limit of detection were assigned a value of 4 ng/L for statistical testing (17 patients). Dynamic hs-cTnT change was calculated by dividing the absolute value of the difference (between the peak and minimum hs-cTnT values) by the minimum hs-cTnT value.

Statistical analysis

Statistical analyses were performed using statistical software SPSS for Windows, version 25.0 (IBM Corp., Armonk, New York, USA) and R, version 3.6.1 (R Foundation for Statistical Computing, Vienna, Austria). Categorical variables are presented as counts and percentages and compared using the Fisher's exact test, or Mantel-Haenszel linear-by-linear association test for trend. Continuous variables are expressed as mean and standard deviation, or median and interquartile range (IQR) as appropriate. Continuous variables were compared between groups using Mann-Whitney U-test.

Multivariable linear regression analysis was performed to evaluate the association between admission HR and peak hs-cTnT levels. Variables with $P<.10$ in univariate analysis were entered into a multivariable regression model using a backward procedure ($P>.10$ for exclusion and $P<.05$ for model inclusion). Variance inflation factors were <1.6 in multivariable regression models ensuring lack of multicollinearity between variables. Generalized additive model, with scaled t distribution and identity link, was used to explore nonlinear relationships between admission HR (as thin-plate spline) and peak hs-cTnT (response variable) using *gam* in the *mgcv* package. Adjustments were made, based on the multiple linear regression results, for age, hemoglobin, estimated glomerular filtration rate, diabetes mellitus, congestive heart failure, new-onset AF and palpitation symptoms. The restricted maximum likelihood criterion was used for smoothing selection. The Akaike information criterion (AIC) was used to assess performance of linear and generalized additive models.

Finally, patients were divided into 5 groups of equal size based on their admission HR (rounded to the nearest 5 beats per minute [bpm]): <90, 90-109, 110-124, 125-139, and ≥140 bpm. Multivariable logistic regression analysis was used to evaluate the association between groups of patients with different HRs and elevated hs-cTnT level (>14 ng/L). Adjustments were made for age, hemoglobin, estimated glomerular filtration rate, diabetes mellitus, congestive heart failure, new-onset AF and palpitation symptoms. P value <.05 was considered statistically significant.

© 2020 Pouru JP et al. *JAMA Network Open.*
